# "Near-miss" obstetric events and maternal deaths in Sagamu, Nigeria: a retrospective study

**DOI:** 10.1186/1742-4755-2-9

**Published:** 2005-11-01

**Authors:** Olufemi T Oladapo, Adewale O Sule-Odu, Adetola O Olatunji, Olusoji J Daniel

**Affiliations:** 1 Maternal and Fetal Health Research Unit, Department of Obstetrics and Gynaecology, Obafemi Awolowo College of Health Sciences/ Olabisi Onabanjo University Teaching Hospital, Sagamu, Ogun State, Nigeria; 2 Department of Community Medicine and Primary care, Obafemi Awolowo College of Health Sciences/ Olabisi Onabanjo University Teaching Hospital, Sagamu, Ogun State, Nigeria

## Abstract

**Aim:**

To determine the frequency of near-miss (severe acute maternal morbidity) and the nature of near-miss events, and comparatively analysed near-miss morbidities and maternal deaths among pregnant women managed over a 3-year period in a Nigerian tertiary centre.

**Methods:**

Retrospective facility-based review of cases of near-miss and maternal death which occurred between 1 January 2002 and 31 December 2004. Near-miss case definition was based on validated disease-specific criteria, comprising of five diagnostic categories: haemorrhage, hypertensive disorders in pregnancy, dystocia, infection and anaemia. The near-miss morbidities were compared with maternal deaths with respect to demographic features and disease profiles. Mortality indices were determined for various disease processes to appreciate the standard of care provided for life-threatening obstetric conditions. The maternal death to near-miss ratios for the three years were compared to assess the trend in the quality of obstetric care.

**Results:**

There were 1501 deliveries, 211 near-miss cases and 44 maternal deaths. The total near-miss events were 242 with a decreasing trend from 2002 to 2004. Demographic features of cases of near-miss and maternal death were comparable. Besides infectious morbidity, the categories of complications responsible for near-misses and maternal deaths followed the same order of decreasing frequency. Hypertensive disorders in pregnancy and haemorrhage were responsible for 61.1% of near-miss cases and 50.0% of maternal deaths. More women died after developing severe morbidity due to uterine rupture and infection, with mortality indices of 37.5% and 28.6%, respectively. Early pregnancy complications and antepartum haemorrhage had the lowest mortality indices. Majority of the cases of near-miss (82.5%) and maternal death (88.6%) were unbooked for antenatal care and delivery in this hospital. Maternal mortality ratio for the period was 2931.4 per 100,000 deliveries. The overall maternal death to near-miss ratio was 1: 4.8 and this remained relatively constant over the 3-year period.

**Conclusion:**

The quality of care received by critically ill obstetric patients in this centre is suboptimal with no evident changes between 2002 and 2004. Reduction of the present maternal mortality ratio may best be achieved by developing evidence-based protocols and improving the resources for managing severe morbidities due to hypertension and haemorrhage especially in critically ill unbooked patients. Tertiary care hospitals in Nigeria could also benefit from evaluation of their standard of obstetric care by including near-miss investigations in their maternal death enquiries.

## Background

For many years, evaluation of maternal healthcare services aimed at improving the quality of obstetric care has traditionally relied on enquiries into maternal deaths. More recently, review of cases at the very severe end of the maternal morbidity spectrum, described as "near-miss" (those who nearly died), has been found to be a useful complement to investigation of maternal mortality [1,2]. Review of near-miss cases has the potential to highlight the deficiencies as well as the positive elements in the provision of obstetric services in any health system. Unlike in the developed countries, there is limited experience with the use of near-miss reviews as a tool for monitoring the quality of maternity services in developing countries. This is probably as a result of the persistently high levels of maternal mortality that has overshadowed other severe obstetric complications, from which lessons could equally be learned. In spite of the high maternal mortality ratios in many of the centres in resource-poor settings, the actual number of maternal deaths per centre may not allow detailed quantification of associated risk factors and determinants that are locally important. Because near-misses occur much more frequently than maternal deaths, more comprehensive and statistically reliable quantitative analyses that are of value to clinical audit can be rapidly conducted [1-4]].

At Olabisi Onabanjo University Teaching Hospital, Sagamu, a state-owned referral centre in southwest Nigeria, maternal mortality ratio is close to 2000 per 100,000 deliveries [5]. The majority of these maternal deaths are largely unpreventable as they occur in unbooked emergency cases that present too late to the hospital and die shortly after admission. Therefore, isolated enquiry into maternal deaths in this centre is unlikely to yield adequate information when the focus of investigation is on the standard of in-hospital care. As shown in previous studies, inclusion of near-miss review in maternal death enquiry can better inform the quality of obstetric care at different levels of healthcare delivery at more frequent intervals [3,4,6].

In order to provide an insight into the quality of maternal care provided in our institution, we embarked on a retrospective study to determine the frequency of near-miss (severe acute maternal morbidity) and the nature of near-miss events, and comparatively analysed near-miss morbidities and maternal deaths among pregnant women managed in this centre over a 3-year period. The review is expected to serve as a complementary method for auditing the quality of maternal healthcare in this institution.

## Methods

### Hospital Setting

The study was conducted at the obstetric unit of Olabisi Onabanjo University Teaching Hospital (OOUTH), Sagamu, a publicly funded tertiary care institution, which serves as the major referral centre for other public and private hospitals within Ogun State, in southwest Nigeria. In addition to providing emergency obstetric services to women referred from other centres, the hospital also provides antenatal care and delivery services for both unreferred low and high-risk pregnant women from Sagamu community and neighbouring towns. The centre provides emergency obstetric and gynaecological care 24 hours a day. Although patients are expected to pay for their services, in emergency situations, they are managed within the means of existing resources before funds are made available. Nine consultant obstetricians and an average of 15 registrars and 30 midwives ran the three obstetric units of the hospital during the reviewed period. The hospital provides blood transfusion services from limited stock and relatives of patients are requested to donate or pay for blood when needed. Each unit of blood costs between N1500-N2000 (approximately £6–10) during the reviewed period. The only intensive care unit (ICU) of the hospital is within the main surgical theatre though patients requiring critical care are admitted from other units after paying a certain fee. Proliferation of many private hospitals and traditional birth homes within Ogun State in recent past has limited the total number of deliveries but has relatively increased the frequency of complicated pregnancies and deliveries managed in the obstetric unit. An average of 40–45 deliveries are conducted per month out of which 10–15 are unbooked.

### Definition of cases

Near-miss events are defined as acute obstetric complications that immediately threaten a woman's survival but do not result in her death either by chance or because of hospital care she receives during pregnancy, labour or within 6 weeks after termination of pregnancy or delivery [1] while a near-miss case is a woman with at least one near-miss event. For identifying near-miss events, we applied the disease-specific criteria that were employed by Filippi et al [7] in similar hospital settings in West Africa, which are based on five main diagnostic groups. These are haemorrhage (leading to shock, emergency hysterectomy, coagulation defects and/or blood transfusion of ≥ 2 litres); hypertensive disorders in pregnancy (eclampsia and severe pre-eclampsia with clinical/laboratory indications for termination of pregnancy to save the woman's life [8]); dystocia (uterine rupture and impending rupture e.g. prolonged obstructed labour with a previous caesarean section); infection (hyperthermia or hypothermia or a clear source of infection and clinical signs of septic shock) and anaemia (low haemoglobin level: haematocrit < 6 g/dl) or clinical signs of severe anaemia in women without severe haemorrhage.

Life-threatening obstetric conditions refer to maternal complications severe enough to cause near-miss morbidity and maternal death while "critically ill obstetric patients" are women who suffered life-threatening obstetric conditions (i.e. cases of near-miss and maternal death). Maternal death is defined according to the tenth revision of International Classification of Diseases (ICD-10) by the World Health Organization [9].

### Study design and identification of cases

Near-miss cases were retrospectively identified among women with pregnancy-related complications admitted into the obstetric units of the hospital between 1 January 2002 and 31 December 2004. Using the provisional and final diagnoses documented in the admission-discharge register of the hospital, case files of women whose diagnoses met the above pre-defined criteria as well as those with the possibility of being associated with severe acute maternal complications were retrieved for scrutiny by the Near-miss Audit Committee comprising three consultant obstetricians and three specialist registrars. Overall, 520 cases were retrieved for scrutiny. For each case of near miss, data were collected on demographic characteristics including gestational age at the time of sustaining the near-miss morbidity, nature of obstetric complication(s) responsible, presence of organ-system dysfunction/failure, ICU admission, timing of near-miss event with respect to admission, fetal outcome in those associated with labour and length of hospital stay. Information on maternal deaths and deliveries conducted during the reviewed period were obtained from the labour/delivery registers and case files from the Medical Records Department. For each case of maternal death, data were collected on the demographic characteristics including gestational age at the time of death and the underlying cause of death.

### Data analysis

Data were entered into a computer database using Microsoft Excel spreadsheet and statistical analysis was performed with Epi Info 2002 software (CDC and WHO, 2002)[10]. Results are presented as frequencies, percentages and descriptive statistics. The prevalence of near-miss cases is defined as the number of near-miss cases divided by the number of deliveries in the hospital. The frequencies of near-miss events are reported according to the clinical condition responsible, referral status of the patients and whether the complications were present upon arrival or occurred while on admission at the hospital. The near-miss morbidities were compared with maternal mortality for their demographic features and underlying disease processes. In order to appreciate the standard of care provided for each disease process, we calculated the mortality index for each obstetric condition. This was defined as the number of maternal deaths resulting from a particular obstetric condition divided by the sum of the near-miss morbidities and maternal deaths occurring from such obstetric condition, expressed as a percentage. It reflects the proportion of each life-threatening obstetric condition, which ended in maternal death. Maternal mortality ratio was calculated as the number of maternal deaths per 100,000 deliveries.

Categorical variables were compared with the χ^2 ^or Fisher's exact test when appropriate while continuous variables were compared with the Student's *t*-test. Differences between data were considered statistically significant when *p*<0.05.

## Results

During the reviewed period, there were 1501 deliveries, 211 near-miss cases and 44 maternal deaths. As shown in Table 1, the demographic characteristics of women who sustained near-miss complications and those who died are comparable. At least half of the women in each group were within the ages of 21 and 30 years. Over one-third of women in each group were nulliparous, in keeping with the parity demographics of our obstetric population.

**Table 1:** A comparison of the demographic characteristics of women with near-miss morbidity and maternal death.

	Near-miss cases	Maternal deaths
	n = 211	n = 44
**Age (years)**		
≤ 20	**24 (11.4)**	**6 (13.6)**
21–25	**58 (27.5)**	**9 (20.5)**
26–30	**56 (26.5)**	**13 (29.5)**
31–35	**47 (22.3)**	**8 (18.2)**
>35	**26 (12.3)**	**8 (18.2)**
Range	**16–44**	**18–45**
Mean ± SD	**28.1 ± 6.1**	**28.6 ± 5.6**
**Parity**		
0	**75 (35.5)**	**15 (34.1)**
1–4	**113 (53.6)**	**25 (56.8)**
≥ 5	**23 (10.9)**	**4 (9.1)**
Range	**0–7**	**0–7**
Median	**2**	**1**
**Booking status**		
Unbooked at OOUTH	**174 (82.5)**	**39 (88.6)**
**Gestational age (weeks)**		
<13	**26 (12.3)**	**2 (4.5)**
13–28	**13 (6.2)**	**1 (2.3)**
>28	**172 (81.5)**	**41 (93.2)**

Percentage in parenthesis

A total of 242 near-miss events were identified among the near-miss cases with frequencies decreasing from 95 in 2002 to 65 in 2004 (Table 2). This implies that 31 women had more than one near-miss morbidity, giving an average of 1.2 near-miss morbidities per case. Table 2 shows that the most common types of near-miss events fall under the diagnostic categories of hypertensive disorders in pregnancy, haemorrhage and dystocia. Hypertensive disorders in pregnancy and haemorrhage were responsible for 61.6 % of all near-miss events. Most events of near-miss due to haemorrhage developed in the later part of pregnancy with 41.1% occurring postpartum. Haemorrhage due to abortion did not cause any near-miss complication over the 3-year period. Near-miss events related to infection and anaemia were the least common.

**Table 2:** Diagnosis distribution and trend of near-miss events in Sagamu, Nigeria

Criteria	**2002**	**2003**	**2004**	**Total**
**Haemorrhage**	26 (28.3)	30 (35.3)	17 (26.2)	73 (30.2)
*Early pregnancy*	6(6.5)	9 (10.6)	9 (13.8)	24 (9.9)
				
Ectopic pregnancy	6	9	9	24
Abortion	0	0	0	0
				
*Late pregnancy*	20 (21.7)	21 (24.7)	8 (12.3)	49 (20.2)
Placenta praevia	4	2	2	8
Abruptio placentae	5	4	2	11
Postpartum haemorrhage	11	15	4	30
Others	0	0	0	
				
**Hypertension**	32 (34.8)	21 (24.7)	23 (35.4)	76 (31.4)
Eclampsia	22	8	9	39
Severe preeclampsia	10	13	14	37
				
**Dystocia**	17 (18.5)	19 (22.4)	11 (16.9)	47 (19.4)
Uterine rupture	3	4	3	10
Impending rupture	14	15	8	37
				
**Infections**	9 (9.8)	5 (5.9)	6 (9.2)	20 (8.3)
				
**Anaemia**	8 (8.7)	10 (11.8)	8 (12.3)	26 (10.7)
				
All near-miss events	92	85	65	242 (100.0)

Percentage in parenthesis

The various causes of maternal deaths between 2002 and 2004 are shown in Table 3. Forty-two (95.5%) of the deaths were direct maternal deaths while 2 (4.5%) were indirect maternal deaths. Maternal deaths were also most commonly due to hypertensive disorders in pregnancy and haemorrhage both responsible for 50.0% of all deaths. Overall, eclampsia was the leading cause of deaths singly accounting for 22.7% of all maternal deaths. Most (8/9) of the deaths due to haemorrhage were cases of postpartum haemorrhage. HIV infection (2.3%) and septic abortion (2.3%) were uncommon causes of maternal deaths during the reviewed period.

**Table 3:** Causes of maternal deaths in Sagamu, Nigeria (2002–2004)

**Causes**	n = 44	(%)
**Haemorrhage**	**9**	**20.5**
*Early pregnancy*		
Ectopic pregnancy	1	2.3
Abortion	-	-
		
*Late pregnancy*		
Placenta praevia	-	-
Abruptio placentae	-	-
Postpartum haemorrhage	8	18.2
		
**Hypertension**	**13**	**29.5**
Eclampsia	10	22.7
Severe preeclampsia	3	6.8
		
**Dystocia**	**6**	**13.6**
Uterine rupture	6	13.6
		
**Infections**	**8**	**18.2**
Puerperal sepsis	2	4.5
Chorioamnionitis	4	9.1
Septic abortion	1	2.3
*HIV infection	1	2.3
**Anaemia **(not due to haem- orrhage	**5**	**11.4**
Pulmonary embolism	1	2.3
Anaesthetic complication	1	2.3
*Pre-existing medical disease	1	2.3

*Indirect obstetric causes

In Table 4, the disease profile of near-miss morbidities was compared with that of maternal mortality. Besides infectious morbidity, the categories of maternal complications responsible for near-miss and maternal mortality followed the same order of decreasing frequency (viz hypertensive disorders in pregnancy, haemorrhage, dystocia and anaemia). Significantly more women died after developing severe morbidity due to uterine rupture and infection. The leading life-threatening obstetric conditions were hypertensive disorders in pregnancy (31.4%) and haemorrhage (29.0%). Uterine rupture (37.5%) and infection (28.6%) had the highest mortality indices though they were less frequent life-threatening obstetric conditions. The lowest mortality indices were recorded for severe morbidities associated with haemorrhage in early pregnancy and antepartum haemorrhage.

**Table 4:** A comparison of near-miss events and primary causes of maternal deaths

**Complication**	**NME**	*Maternal deaths	***P***	**LTOC (%)**	**Mortality index (%)**
	n (%)	n (%)		n (%)	
**Haemorrhage**	73 (30.2)	9 (22.0)	0.284	82 (29.0)	11.0
					
*Early pregnancy*	24 (9.9)	1 (2.4)	0.145	25 (8.8)	4.0
Ectopic pregnancy	24	1	0.145	25 (8.8)	4.0
Abortion	0	0		0.0 (0.0)	0.0
					
*Late pregnancy*	49 (20.2)	8 (19.5)	0.914	57 (20.1)	14.0
Placenta praevia	8	0	0.608	8 (2.8)	0.0
Abruptio placentae	11	0	0.376	11 (3.9)	0.0
Postpartum haemorrhage	30	8	0.217	38 (13.4)	21.1
					
**Hypertension**	76 (31.4)	13 (31.7)	0.969	89 (31.4)	14.6
Eclampsia	39	10	0.195	49 (17.3)	20.4
Severe pre-eclampsia	37	3	0.175	40 (14.1)	7.5
					
**Dystocia**	47 (19.4)	6 (14.6)	0.467	53 (18.7)	11.3
Uterine rupture	10	6	0.017	16 (5.7)	37.5
Impending rupture	37	0	0.007	37 (13.1)	0.0
					
**Infection**	20 (8.3)	8 (19.5)	0.042	28 (9.9)	28.6
					
**Anaemia**	26 (10.7)	5 (12.2)	0.787	31 (11.0)	16.1
					
Total	242 (100.0)	41 (100.0)		283 (100.0)	14.5

*Excluding anaesthetic death (n = 1), pulmonary embolism (n = 1) and pre-existing medical disease (n = 1)

Mortality index = maternal deaths divided by life-threatening obstetric conditions

LTOC: life-threatening obstetric condition

NME: near-miss events

Table 5 shows that there was a significant fall in the prevalence of near-miss cases from 2002 to 2004 (χ^2 ^= 9.01, 2df; p = 0.011) with an overall prevalence of 140.6 per 1000 deliveries. Critically ill obstetric patients constituted 17.0% of all women who delivered during the reviewed period. The frequency of critically ill obstetric patients also decreased significantly between 2002 and 2004 (p = 0.002). Maternal mortality ratio for the 3-year period was 2931.4 per 100,000 deliveries. Though there were fewer deaths in 2004, there was no significant difference between the maternal mortality ratios of 2002 and 2004 (p = 0.236). The overall maternal death to near-miss ratio was 1: 4.8 with no significant difference in this relationship between the years of study. Overall, 17.3% of critically ill obstetric patients died during the 3-year period. Majority (80.6%) of the near-miss cases were referred from other facilities namely traditional birth attendant homes, primary and secondary healthcare units and private hospitals within Ogun State and beyond. Most near-miss cases already had near-miss morbidity upon arrival at OOUTH, Sagamu while only 15.6% of them became near-miss after admission to the hospital. The proportion of near-miss events occurring after admission varied between diagnostic categories; haemorrhage (23.3%), hypertensive disorders (15.8%), dystocia (8.5%), infections (10.0%) and anaemia (11.5%).

**Table 5:** Frequency and characteristics of near-miss cases and maternal death to near-miss ratios for 2002–2004.

	**2002**	**2003**	**2004**	**Total**
Deliveries (n)	475	545	481	1501
Live births	433	502	443	1378
Near-miss cases (n)	85	71	55	211
*Referred from other facility *[n (%)]	68 (80.0)	58 (81.7)	44 (80.0)	170 (80.6)
*On arrival *[n (%)]	71(83.5)	59 (83.1)	49 (89.1)	179 (84.8)
*During hospitalisation*	*15 (17.6)	12 (16.9)	6 (10.9)	33 (15.6)
Near-miss cases per 1000 deliveries	178.9	130.3		114.3 140.6
*On arrival*	149.5	108.3	101.9	119.3
*During hospitalisation*	31.6	22.0	12.5	22.0
Maternal deaths (n)	17	16	11	44
MMR/100,000 deliveries	3578.9	2935.8	2286.9	2931.4
Critically ill obstetric patients	102	87	66	255
Maternal death to near-miss ratio	1: 5	1: 4.4	1: 5	1: 4.8

MMR: maternal mortality ratio

*One patient qualified as a near-miss on arrival and during hospitalisation

Only 9 (4.3%) of the near-miss cases were managed in the ICU while organ-system dysfunction/failure were recorded in 19 (9.0%) of them. The nature of organ-system dysfunction/failure and the associated obstetric factors among the near-miss cases are shown in Table 6. The two most commonly affected organ-systems were the renal and vascular systems. Among the 167 near-miss cases that were associated with labour, 37.7% and 6.5% resulted in stillbirths and early neonatal deaths, respectively. Duration of hospital stay for near-miss cases ranged between 2 and 74 days (median 11 days, interquartile range: 8–15 days).

**Table 6:** Causes of organ-system dysfunction/failure in near-miss cases

**Organ-system**	n = 19*	**Obstetric causes (n)**
Cardiac (pulmonary oedema)	3	Severe pre-eclampsia (2)
		Severe anaemia (unrelated to haemorrhage (1)
Coagulation	1	Abruptio placentae
Renal	7	Eclampsia (4)
		Severe pre-eclampsia (1)
		Septic abortion (1)
		Postpartum haemorrhage (1)
Vascular	7	Ruptured ectopic pregnancy (1)
		Uterine rupture (1)
		Postpartum haemorrhage (5)
Cerebral	4	Eclampsia (4)
Immunologic	1	HIV-related sepsis (1)

*Note that some women had >1 organ-system dysfunction/failure

## Discussion

The need to assess the quality of obstetric care in any centre is paramount to understanding the improvement resulting from investment in its maternity services. Up till now, evaluation of obstetric performance in many Nigerian hospitals is limited to investigations of maternal deaths, an indicator that is vulnerable to many flaws in this environment. To the best of our knowledge, ours is the first review in this country that quantitatively examined the quality of obstetric care using alternative indices.

The study shows that severe acute maternal morbidities (near-miss) occur in a considerable percentage of women managed in this obstetric unit. Life-threatening obstetric conditions, including those that resulted in deaths complicated up to 17% of all deliveries during the reviewed period. This implies that obstetricians in this centre were confronted with life-saving emergency situations in almost 1 out of every 6 women who utilised their obstetric services. While the prevalence of our near-miss cases shows some degree of consistency with the reports from other teaching hospitals in West Africa [7], it is several-folds higher than those published from developed countries [11,12]. This disparity is possibly due to differences in definition and identification of cases, which are major limitations in comparison of near-miss data across institutions [13,14]. Studies in industrialised countries commonly use ICU admission or organ-system dysfunction/failure as their criteria for case selection [11,15]. Though organ-system based criteria are regarded as the most specific and least vulnerable to bias [13], we adopted a tested case definition that best fits the circumstances in our environment to allow local improvement in services and comparison of studies in our setting. We conclude that the wide difference in the magnitude of our cases compared to those quoted in high-resource settings is unlikely to be due to overestimation of our near-miss cases since what constitutes near-miss morbidity in any centre is dependent on contextual factors and the figures only depicted the number of women at the verge of dying in their respective prevailing circumstances. This conclusion is further supported by the fact that our near-miss cases were approximately five times as frequent as maternal deaths, similar to the findings in studies that used organ-system based criteria [16].

One of the advantages of the criteria used for our case definition is that it mirrors the major causes of maternal mortality and therefore readily permits comparison that allows assessment of the standard of care with respect to common causes of maternal deaths. As logically expected, there were no major differences in the underlying pathology for near-miss morbidities and maternal deaths indicating that near-miss review can be a useful surrogate of maternal death analysis in this centre. Similar to the findings in many previous studies [7,11,15], hypertensive disorders and haemorrhage were the leading causes of near-miss morbidities accounting for almost two-thirds of all cases. The contribution of these complications to maternal deaths, however, signifies a poor response of our system to modify the major disease profile of its obstetric population. In view of the referral status of most critically ill women, reduction of maternal deaths in this centre therefore requires channelling of resources towards the prevention of haemorrhage and hypertensive disorders at the lower levels of healthcare while strengthening the resources for their treatment in the teaching hospital.

The importance of including near-miss investigations in maternal death audits is demonstrated by this review. Comparison of the disease processes responsible for near-miss and maternal deaths shows that infection, with a mortality index of 28.6%, constituted a significant threat to the survival of affected patients though it was the least frequent cause of life-threatening obstetric conditions. Similarly, it became clear that uterine rupture received the poorest form of care even though it accounted for 5.7% of life-threatening obstetric conditions. The level of care provided for pregnancies complicated by eclampsia also deserves special attention. According to the mortality index, approximately one-fifth of critically ill eclamptic patients died in this centre. Though eclampsia is a known major cause of maternal death worldwide, the poor standard of care for women with eclampsia in our unit may be related to the existing management policy for hypertensive disorders in pregnancy. Up till now, magnesium sulphate, which has been shown to reduce eclampsia-related risk of maternal mortality [17,18], is yet to be adopted for use in this institution. In the light of our findings, efforts to reduce maternal death from hypertensive disorders must include urgent adoption of a clear and up-to-date evidence-based protocol for treating eclampsia.

The management of haemorrhage due to abortion during the reviewed period is commendable as none of such cases caused near-miss morbidity or maternal mortality. Though this may be related to the frequent training of members of staff in manual vacuum aspiration and incorporation of postabortal care into the existing management protocol of abortion in the last five years, it is possible that affected women did not present at the hospital either because they did not survive or because of fear of legal prosecution in cases of criminal abortion. Of the various types of life-threatening obstetric haemorrhage, postpartum haemorrhage constituted the greatest danger to affected women while early pregnancy haemorrhagic complications and antepartum haemorrhage were less risky. This implies that efforts need to be focussed on improving the protocols and resources for combating postpartum haemorrhage, while maintaining and improving the existing preventive measures and treatment strategies for early pregnancy complications and antepartum haemorrhage.

As shown in this study, the lack of proper antenatal care is a major determinant of adverse maternal outcome. Majority of the women with near-miss morbidity arrived at the hospital in critical condition having being referred from both modern and traditional maternity facilities. Though some authors suggest that near-miss upon arrival at the hospital should not be used to assess the quality of care at the admitting facility [7], we believe that the proportion of referred near-miss cases to our obstetric unit reflected our ability to prevent maternal deaths, even in previously unanticipated situations. What is worrisome, however, is the recorded maternal death to near-miss ratio, a useful indicator of the quality of care received by near-miss cases irrespective of their primary source of antenatal or labour care [13]. A maternal death to near-miss ratio of approximately 1: 5 indicates that for every 5 women who survived life-threatening complications in this centre, one maternal death was also recorded. This ratio, which reflects the overall standard of obstetric care, is poorer than 1: 11–22 reported from similar centres in Niger [19], Cote d'Ivoire and Benin [7] respectively and a far cry from the 1: 117–223 reported in Europe [12-14] using the same criteria for case definition.

It is unlikely that the overall substandard level of care in this centre is due to a higher prevalence of life-threatening complications compared to other centres. This is because with the recorded delivery rate, the prevalence of critically ill obstetric patients translates to an average of 7 of such patients being managed per month (255/36 months) and a maternal death to near-miss ratio of 1: 5 is unjustifiable with the existing human resources. Though this level of care could be attributed to other extraneous factors ranging from cost of obstetric services, mismanagement at the sources of referral to lapses in the referral chain, it is the duty of a referral hospital to maintain a good standard of care if the utilisation of obstetric services among the population is to be encouraged. In spite of the decreasing trend in the frequency of near-miss cases over the 3 years, the similarity in the death to near-miss ratios indicates that there was no significant improvement in the level of care over these years. Therefore, the significant fall in the prevalence of near-miss cases is probably a reflection of recent governmental efforts to improve obstetric services at the primary and secondary healthcare units, which are the main sources of near-miss cases managed at the teaching hospital.

Some important issues concerning definitions in near-miss studies were also illustrated in this investigation. It appears that identification of cases based on ICU admission or organ-system failure/dysfunction as used in some studies may underestimate the frequency of severe maternal morbidities in our setting since they occurred in only 4.3% and 9.0% of all near-miss cases, respectively. Though data collection is easy, a major disadvantage of using ICU admission as the criteria for case selection is that it is dependent on factors such as availability, capacity and location of ICU and institutional guidelines for ICU admission [1]. Therefore, such method is unlikely to produce accurate data in a centre like ours where ICU is not available in the labour ward unit and patients can only be admitted to the general ICU after payment of certain fees. Likewise, comparison of the frequencies of women identified to have suffered organ dysfunction with those who died is inconsistent with the usual relationship between near-misses and maternal deaths suggesting that organ dysfunction was probably poorly documented. This problem is most likely related to our retrospective identification of cases which essentially relied on obstetric diagnoses indexed in the admission-discharge register. Cases of organ-system dysfunction are best detected as they occur, and are therefore more reliably identified in prospective studies.

A major limitation of this study is its retrospective nature. Besides the possibility of underestimating the near miss-cases as a result of incomplete documentation in case files, the methodology also discouraged assessment of substandard care with respect to the health workers or health administration and patient-orientated missed opportunities. Evaluation of the circumstances surrounding near-misses and maternal deaths would shed light on avoidable factors and therefore enable more focussed remedial actions. This aspect needs to be considered in subsequent near-miss investigations in this institution.

## Conclusion

In summary, our review shows that besides the 44 women who died due to pregnancy-related complications, there were 211 additional women who received critical care during the same period supporting the view that near-miss appraisal provides a larger sample to assess the threat to maternal life. The overall maternal death to near-miss ratio, however, indicates that a significant proportion of critically ill women died, suggesting a suboptimal level of care for life-threatening complications. Since there were little differences in the underlying disease processes causing near-miss and maternal mortality, evaluation of the circumstances surrounding near-miss cases could act as a proxy for maternal death in this centre. Efforts geared towards improvement in the management of near-miss morbidities would definitely go a long way in reducing the present maternal mortality ratio. From the findings of this review, attempts to reduce maternal deaths may best be achieved by developing evidence-based protocols for the management of severe hypertension and haemorrhage especially for critically ill referred patients. In addition, considerable efforts should be made to improve maternal care for infrequent but important life-threatening obstetric conditions such as uterine rupture and infection. Necessary facilities should be made available and training of personnel and emergency drills should be frequently conducted to combat the identified disease processes that received suboptimal care. Although this study did not specifically address avoidable factors, it has nevertheless raised awareness of the deficiencies in the management of serious maternal illnesses. It is apparent from this review that tertiary institutions in Nigeria could also benefit from evaluation of their quality of obstetric care by including near-miss investigations in their maternal death enquiries.

## Competing interests

The author(s) declare that they have no competing interests.

## Authors' contributions

OOT conceived and designed the study. OOT drafted the manuscript while AOS and AOO critically revised it for intellectual contents. OOT, AOS and AOO were members of the committee that collected the data. OOT and OJD analysed and interpreted the data.

## References

[B1] Ronsmans C, Filippi V (2004). Reviewing severe maternal morbidity: learning from survivors from life-threatening complications. Beyond the Numbers: Reviewing Deaths and Complications to Make Pregnancy Safer.

[B2] Pattinson RC, Buchmann E, Mantel G, Schoon M, Rees H (2003). Can enquiries into severe acute maternal morbidity act as a surrogate for maternal death enquiries?. BJOG.

[B3] Cochet L, Pattinson RC, MacDonald AP (2003). Severe acute maternal morbidity and maternal death audit- a rapid diagnostic tool for evaluating maternal care. S Afr Med J.

[B4] Vandecruys HIB, Pattinson RC, Macdonald AP, Mantel GD (2002). Severe acute maternal morbidity and mortality in the Pretoria Academic Complex: changing patterns over 4 years. Eur J Obstet Gynecol Reprod Biol.

[B5] Sule-Odu AO (2000). Maternal deaths in Sagamu, Nigeria. Int J Gynecol Obstet.

[B6] Gandhi MN, Welz T, Ronsmans C (2004). Severe acute maternal morbidity in rural South Africa. Int J Gynecol Obstet.

[B7] Filippi V, Ronsmans C, Gohou V, Goufodji S, Lardi M, Sahel A, Saizonou J, De Brouwere V (2005). Maternity wards or emergency obstetric rooms? Incidence of near-miss events in African hospitals. Acta Obstet Gynecol Scand.

[B8] Robson SC, Edmond DK (1999). Hypertension and renal disease in pregnancy. Dewhurst's Textbook of Obstetrics and Gynaecology for Postgraduates.

[B9] World Health Organization (1993). ICD-10: International statistical classification of diseases and health-related problems. Tenth Revision.

[B10] Centers for Disease Control and Prevention (CDC) and World Health Organization (2002). Epi Info 2002 – Database and statistics software for public health professionals.

[B11] Baskett TF, Sternadel J (1998). Maternal intensive care and near-miss mortality in obstetrics. BJOG.

[B12] Waterstone M, Bewley S, Wolfe C (2001). Incidence and predictors of severe obstetric morbidity: case-control study. BMJ.

[B13] Say L, Pattinson RC, Gülmezoglu AM (2004). WHO systematic review of maternal morbidity and mortality: the prevalence of severe acute maternal morbidity (near miss). Reprod Health.

[B14] Minkauskiene M, Nadisauskiene R, Padaiga Z, Makari S (2004). Systematic review on the incidence and prevalence of severe maternal morbidity. Medicina.

[B15] Murphy DJ, Charlett P (2002). Cohort study of near-miss maternal mortality and subsequent reproductive outcome. Eur J Obstet Gynecol Reprod Biol.

[B16] Mantel GD, Buchmann E, Rees H, Pattinson RC (1998). Severe acute maternal morbidity: a pilot definition for a near-miss. BJOG.

[B17] Duley L, Henderson-Smart D (2003). Magnesium sulphate versus diazepam for eclampsia. The Cochrane Database of Systematic Reviews.

[B18] Duley L, Gülmezoglu AM, Henderson-Smart DJ (2003). Magnesium sulphate and other anticonvulsants for women with pre-eclampsia. The Cochrane Database of Systematic Reviews.

[B19] Prual A, Huguet D, Gabin O, Rabe G (1998). Severe obstetric morbidity of the third trimester, delivery and early puerperium in Niamey (Niger). Afr J Reprod Health.

